# Adrenomedullin orchestrates treatment resistance in hepatocellular carcinoma via immune microenvironment remodeling

**DOI:** 10.3389/fgene.2025.1721263

**Published:** 2025-12-11

**Authors:** Xixi Gao, Yongliang Sun, Jia Huang, Li Xu, Hanchun Huang, Zhiying Yang

**Affiliations:** China-Japan Friendship Hospital, Beijing, China

**Keywords:** hepatocellular carcinoma, transcatheter arterial chemoembolization, sorafenib, adrenomedullin, single-cell RNA sequencing, tumor immune microenvironment

## Abstract

**Background:**

Hepatocellular carcinoma (HCC) displays marked cellular heterogeneity and immune microenvironment complexity that fundamentally influence transcatheter arterial chemoembolization (TACE) treatment responses and patient outcomes. Deciphering the molecular architecture underlying therapy resistance remains essential for advancing precision oncology in HCC management.

**Methods:**

We integrated four single-cell RNA sequencing cohorts with bulk transcriptomic datasets and longitudinal clinical annotations from The Cancer Genome Atlas Liver Hepatocellular Carcinoma database and Gene Expression Omnibus repositories to perform multidimensional analyses. Computational frameworks including single-cell Phenotype Associated Score (scPAS), high-dimensional weighted gene co-expression network analysis (hdWGCNA), and Single-Cell Regulatory Network Inference and Clustering (SCENIC) were deployed to identify resistance-linked cellular subpopulations and pivotal regulatory modules. Functional validation employed adrenomedullin (ADM)-depleted Huh7 cellular models and xenograft tumor-bearing mouse systems, with mechanistic interrogation via Western immunoblotting, quantitative reverse transcription polymerase chain reaction, and Kaplan-Meier survival estimation to confirm ADM biological functions and clinical relevance.

**Results:**

We identified a TACE-resistant malignant cell subset (scPAS+) characterized by pronounced activation of glycolytic, hypoxic, and epithelial-mesenchymal transition pathways alongside overexpression of resistance-conferring genes including LINC00221, hexokinase 2, and alpha-fetoprotein. This cellular phenotype demonstrated robust associations with TACE non-responsiveness, sorafenib cross-resistance, and abbreviated patient survival. Patient stratification based on scPAS + signature genes delineated two distinct molecular subgroups: the scPAS + -enriched cohort exhibited marked TACE refractoriness, elevated sorafenib failure rates, immunosuppressive microenvironmental architecture, and diminished 5-year survival probability. Mechanistic investigations established ADM as a critical driver orchestrating this resistance phenotype. ADM depletion attenuated Huh7 cell proliferative capacity, migratory potential, and invasive behavior, reduced xenograft tumor burden in murine models, and substantially potentiated sorafenib antitumor efficacy.

**Conclusion:**

This study delineates an ADM-driven TACE-resistant HCC cellular subtype (scPAS+) that functions simultaneously as a prognostic biomarker and actionable therapeutic target for circumventing treatment resistance.

## 1 Introduction

Hepatocellular carcinoma (HCC) represents the most common form of primary liver cancer and remains the third leading cause of cancer-related mortality worldwide, with an estimated incidence exceeding 1 million cases by 2025 (Llovet et al., 2021; Bray et al., 2024). The global disease burden continues to rise, with epidemiological models projecting a 55% increase in incidence by 2040 (Rumgay et al., 2022). The etiologic landscape of HCC is undergoing a significant transition: while chronic hepatitis B and C virus infections remain predominant risk factors in endemic regions, metabolic dysfunction-associated steatotic liver disease (MASLD) has emerged as a rapidly increasing contributor in Western countries, now affecting approximately 25% of the global population (Huang et al., 2021; Singh et al., 2025; Kim et al., 2021).

The therapeutic paradigm for HCC has experienced profound evolution in recent years. For early-stage disease, surgical resection achieves 30%–50% 5-year survival in cirrhotic patients, while liver transplantation offers approximately 90% 5-year survival for candidates meeting Milan criteria (Zheng et al., 2025). The 2022 Barcelona Clinic Liver Cancer (BCLC) classification update incorporated immunotherapy-based combinations and refined intermediate-stage substratification, fundamentally reshaping treatment algorithms (Reig et al., 2022). First-line systemic therapy has shifted from tyrosine kinase inhibitors to immune checkpoint inhibitor-based regimens, with atezolizumab plus bevacizumab demonstrating superior overall survival compared to sorafenib (median OS: 19.2 vs. 13.4 months) in the landmark IMbrave150 trial (Finn et al., 2020; Cappuyns et al., 2025). Alternative combinations including durvalumab plus tremelimumab provide additional therapeutic options for advanced disease (Abou-Alfa et al., 2022).

Transarterial chemoembolization (TACE) remains the cornerstone treatment for intermediate-stage HCC, with contemporary drug-eluting bead formulations achieving median overall survival of 25.0 months and disease control rates of 80%–95% (Jiang et al., 2019; Zhu et al., 2023). Recent landmark trials have established that combining TACE with immunotherapy significantly improves outcomes: the LEAP-012 trial demonstrated median progression-free survival of 14.6 versus 10.0 months for TACE plus pembrolizumab-lenvatinib versus TACE alone, while EMERALD-1 showed benefits with durvalumab-bevacizumab combinations (Kudo et al., 2022; Sangro et al., 2025). However, TACE harbors intrinsic biological limitations that compromise long-term efficacy. The procedure paradoxically induces local hypoxia, upregulating hypoxia-inducible factor-1α (HIF-1α) and vascular endothelial growth factor (VEGF), which activate resistance signaling cascades and promote neo-angiogenesis (Dong et al., 2018). TACE refractoriness—defined by inadequate response after 2–3 consecutive procedures—affects 37%–49% of patients and represents a major clinical obstacle (Zhang et al., 2023).

Despite significant advances in combination therapies, there remains an imperative need to identify actionable molecular targets that predict or overcome TACE non-response. Elucidating the cellular and molecular underpinnings of treatment resistance—particularly the interplay between tumor cell intrinsic adaptation mechanisms and immune microenvironment remodeling—is essential for developing precision-targeted interventions that will ultimately enhance clinical outcomes for patients with intermediate and advanced HCC.

## 2 Methods

### 2.1 Bulk transcriptome data collection and preprocessing

Bulk transcriptome profiling data along with associated clinical annotations were obtained from two primary repositories. The first source was the liver hepatocellular carcinoma dataset from The Cancer Genome Atlas program (TCGA-LIHC; available through https://portal.gdc.cancer.gov), which provides comprehensive genomic and clinical information. The second source comprised multiple validation cohorts from the GEO database, specifically selected for their clinical relevance: GSE104580 contained samples from patients who underwent transcatheter arterial chemoembolization (TACE), GSE109211 included patients treated with sorafenib, while GSE14520, GSE27150, GSE54236, GSE76427, GSE116174, and GSE144269 served as additional validation datasets representing diverse clinical scenarios. For RNA-sequencing data generated through high-throughput platforms, we implemented a standardized preprocessing workflow. The raw count matrices were initially transformed to transcripts per million (TPM) to account for sequencing depth variations across samples. Subsequently, log_2_ transformation was applied to the TPM values, which served to minimize data skewness and stabilize variance across the expression range, thereby ensuring more reliable downstream statistical analyses. Microarray-based expression data required platform-specific processing steps. We performed probe-to-gene mapping to convert platform-specific probe identifiers to standardized gene symbols. In instances where multiple probes were annotated to interrogate the same gene, we calculated the arithmetic mean of these probe intensities to derive a single representative expression value for each gene. Following gene-level aggregation, between-array normalization was executed using the “normalizeBetweenArrays” function implemented in the limma R package (version 3.50.0), which corrects for systematic technical variations across different arrays. The limma package, based on linear models, is well-suited for large-scale data analysis like ours. After differential analysis, we adjusted for multiple comparisons using the Benjamini–Hochberg procedure to control the false discovery rate (FDR). Specifically, we set the FDR threshold at 0.05 to determine gene significance, thereby minimizing the risk of false positives due to multiple comparisons.

### 2.2 Single-cell RNA sequencing data analysis

Single-cell RNA sequencing datasets were retrieved from the Gene Expression Omnibus repository (GEO; accessible at http://www.ncbi.nlm.nih.gov/geo), specifically including accession numbers GSE149614, GSE151530, GSE156625, and GSE202642. These datasets collectively encompassed liver cancer samples from multiple independent cohorts. We employed the Seurat computational framework (version 4.0.4) to perform comprehensive analysis of liver cancer single-cell transcriptomes. The analytical pipeline initiated with loading raw sequencing outputs via the “Read10X” function, which converts the data into a sparse matrix format (dgCMatrix) optimized for computational efficiency when handling large-scale single-cell datasets. Individual Seurat objects corresponding to the four independent datasets were consolidated using the “merge” function to create a unified analytical framework. To prevent cell barcode collisions during integration, we applied the “RenameCells” function, which ensures unique cell identifiers across the merged dataset. Quality control procedures were rigorously implemented to eliminate technical artifacts and low-quality cellular transcriptomes. We deployed the Scrublet (Wolock et al., 2019) algorithm to computationally identify and exclude potential doublet events, which arise from co-encapsulation of multiple cells. Additional filtering criteria were established to remove cells expressing fewer than 100 unique genes, as these likely represent damaged cells or empty droplets. Conversely, genes detected in fewer than 3 cells were excluded to reduce noise from sporadic detection events. Through these stringent quality control measures, we retained 199,856 high-quality cells derived from 75 liver cancer patients for subsequent analyses. Gene expression profiles underwent normalization using the “LogNormalize” methodology with a scaling factor of 10,000, which accounts for differences in total transcript content across individual cells. To identify the most informative genes for downstream analysis, we utilized the “FindVariableFeatures” function to select the top 2000 genes exhibiting the highest cell-to-cell variability, as these genes typically capture the most biologically relevant heterogeneity. The “ScaleData” function was subsequently applied to regress out technical confounding factors, including variations in total unique molecular identifier (UMI) counts and the percentage of mitochondrial gene expression, the latter serving as a proxy for cellular stress or damage. This scaling procedure centers gene expression values and scales variance to unity, facilitating comparability across genes. Principal component analysis (PCA) was performed on the scaled expression matrix of variable genes, with retention of the first 30 principal components to capture the major axes of variation while reducing computational burden. To mitigate batch effects stemming from technical differences across datasets, we applied the Harmony integration algorithm, which aligns cells from different batches in a shared embedding space while preserving biological variation. Following batch correction, we employed Uniform Manifold Approximation and Projection (UMAP) for two-dimensional visualization of the principal component space, which facilitates intuitive exploration of cellular heterogeneity. Unsupervised cell clustering was conducted through a graph-based approach using sequential application of the “FindNeighbors” and “FindClusters” functions. The optimal clustering resolution parameter (0.6) was determined via the “clustree” function, which evaluates clustering stability across multiple resolution values. Cell type identification was accomplished by examining the expression patterns of canonical marker genes specific to known liver cell populations. Following initial broad cell type classification, epithelial cells were isolated as a separate subset for focused downstream investigation, given their central role in hepatocellular carcinoma biology. To identify truly malignant cells, we performed an analysis for each patient using the SCEVAN (De Falco et al., 2023) algorithm. In this process, we used T cells as a reference for normal cells to identify malignant cells based on copy number changes.

### 2.3 Identification of TACE non-response phenotype-related cells using scPAS

To delineate liver cancer cell subpopulations associated with non-response to TACE therapy, we implemented the single-cell Phenotype Associated Score (scPAS) (Xie et al., 2024) computational framework. This analysis leveraged clinical response data from the GSE104580 cohort, which contains detailed TACE treatment outcome information.

We constructed a logistic regression classifier specifically for the epithelial cell compartment, training the model to discriminate between cells from patients exhibiting differential TACE responses. The scPAS algorithm computes cell-level risk scores that quantify the association of each cell’s transcriptional profile with the non-responder (NR) phenotype. Based on the calculated risk score distribution, epithelial cells were stratified into two distinct categories: scPAS + cells (characterized by high risk scores and strong association with TACE resistance) and scPAS- cells (characterized by low risk scores and association with TACE sensitivity).

For validation purposes, the trained scPAS model was applied to independent cohorts using the scPAS.prediction function, which calculates risk scores for cells in external datasets based on the established classification framework.

### 2.4 Weighted gene Co-expression network analysis and transcription factor identification

We utilized the hdWGCNA package (Morabito et al., 2023) to construct a weighted gene co-expression network specifically tailored for single-cell transcriptome data from the epithelial compartment. This approach identifies groups of genes exhibiting coordinated expression patterns across cells, which often reflect shared biological functions or regulatory mechanisms. The network construction process employed an unsupervised clustering strategy based on topological overlap, ultimately partitioning genes into 20 distinct modules through the dynamic tree cutting algorithm. This algorithm adaptively determines module boundaries by evaluating dendrogram structure and topological properties. The spatial distribution and expression patterns of genes within each module across the single-cell landscape were visualized using heatmap representations, enabling assessment of module-specific cellular expression domains. To identify modules functionally relevant to TACE resistance, we computed Pearson correlation coefficients between each module’s eigengene (the first principal component of module gene expression) and the scPAS+/-phenotype classification. The module demonstrating the strongest positive correlation with scPAS + cells was selected as containing genes potentially driving the TACE-resistant phenotype. Transcription factor activities were quantified using the Single-Cell rEgulatory Network Inference and Clustering (SCENIC) (Aibar et al., 2017) analytical framework (version 1.3.1). This multi-step approach begins with GENIE3 (version 1.26.0), a random forest-based algorithm that infers potential transcription factor-target gene relationships from co-expression patterns across cells. The initial GENIE3-derived network undergoes refinement through integration of cis-regulatory motif information. Specifically, target genes are retained only if their promoter or enhancer regions contain binding motifs corresponding to the predicted transcription factor, as determined through motif enrichment analysis. This integrated approach generates high-confidence transcriptional regulons, defined as sets of genes directly regulated by specific transcription factors through verified cis-regulatory motifs. The activity of each regulon across individual cells was quantified using the AUCell algorithm (version 1.26.0). This method ranks genes by their expression level within each cell and calculates the area under the recovery curve for regulon member genes, producing a continuous activity score that reflects transcription factor regulatory output in each cell.

### 2.5 Cell-cell communication analysis

To systematically characterize cell-cell communication patterns within the liver cancer microenvironment, we employed two complementary computational approaches. The primary analysis utilized CellChat (version 1.6.1) (Jin et al., 2021), a probabilistic framework that infers intercellular signaling based on known ligand-receptor interaction databases. The CellChat analytical workflow commenced with preprocessing of the normalized single-cell expression matrix, followed by grouping of cells according to their annotated cell type identities. Using the CellChatDB.human ligand-receptor database as reference, we identified significantly overexpressed ligands and cognate receptors within each cell population compared to background expression levels. Communication probability for each ligand-receptor pair between cell type pairs was computed using a mass action-based model that considers both expression levels and signaling cofactors. This generates a weighted directed network where edge weights represent the probability and strength of intercellular signaling along specific pathways. CellChat further aggregates individual ligand-receptor interactions into broader signaling pathway categories, enabling systems-level interpretation of communication patterns. The algorithm quantifies both the number of unique interactions and their cumulative signaling strength for each cell type pair.

### 2.6 Functional enrichment analysis

Functional enrichment analysis was performed using the clusterProfiler R package (Wu et al., 2021) (version 4.12.0) to identify biological processes, molecular functions, and pathways over-represented among differentially expressed gene sets. Enrichment testing was conducted against multiple annotation databases including Gene Ontology (GO) and the Kyoto Encyclopedia of Genes and Genomes (KEGG). Statistical significance was assessed using hypergeometric testing with Benjamini–Hochberg correction for multiple hypothesis testing. Gene sets or pathways with false discovery rate (FDR)-adjusted p-values below 0.05 were designated as significantly enriched and retained for biological interpretation. For bulk RNA-sequencing datasets, we employed single-sample Gene Set Enrichment Analysis (ssGSEA) to quantify pathway-level activity scores for individual samples. This approach enables assessment of pathway enrichment within single samples without requiring phenotypic comparison groups. The analysis was executed using the GSVA R package with default parameter settings, taking as input normalized gene expression matrices and curated pathway gene sets from MSigDB collections. For single-cell RNA-sequencing data, pathway activity inference was performed using the AUCell algorithm, which computes enrichment scores based on the recovery of gene set members in the ranked expression profile of each cell. Specifically, genes in each cell are ranked by expression level, and the Area Under the Curve is calculated for the recovery of pathway gene set members in this ranking. Higher AUC values indicate greater enrichment of the gene set in that cell, reflecting elevated pathway activity. All AUCell analyses were conducted using default parameters provided by the AUCell R package (version 1.26.0), ensuring consistency with transcription factor activity quantification described earlier.

### 2.7 Cell line and culture system

The human hepatocellular carcinoma cell line Huh7 was obtained from Procell Life Science and Technology Co., Ltd. Cells were maintained in high-glucose Dulbecco’s Modified Eagle Medium (DMEM, HyClone) supplemented with 10% fetal bovine serum (FBS, Gibco) and cultured in a humidified incubator maintained at 37 °C with 5% CO_2_ atmosphere. Medium replacement was performed at 3-day intervals, and cells in logarithmic growth phase with stable passage history were utilized for all subsequent experiments.

### 2.8 Generation of sh-ADM lentiviral constructs and stable cell line establishment

Short hairpin RNA (shRNA) sequences targeting the ADM gene and corresponding negative control sequences (sh-NC) were designed and cloned into lentiviral vectors by Shanghai GeneChem Co., Ltd. Huh7 cells were seeded in 6-well plates and allowed to reach 50% confluence before transduction with sh-ADM or sh-NC lentiviral particles at a multiplicity of infection (MOI) of 10, supplemented with 5 μg/mL Polybrene to enhance transduction efficiency. Following a 48-h incubation period, cells were subjected to selection using medium containing 2 μg/mL Puromycin. After 2 weeks of continuous selection, stably transfected ADM-knockdown cell lines (sh-ADM group) and negative control cell lines (sh-NC group) were successfully established.

### 2.9 CCK-8 proliferation assessment

Cells from the sh-ADM group, sh-NC group, and sorafenib combination treatment groups were plated in 96-well plates at a density of 2 × 10^3^ cells per well, with triplicate wells established for each experimental condition. Following overnight attachment, 20 μL of CCK-8 solution (5 mg/mL, Dojindo) was added to each well and incubated for an additional 4 h. Absorbance values (optical density, OD) were measured at 450 nm wavelength using a microplate reader (Thermo Fisher). Measurements were performed over 5 consecutive days to generate cell proliferation curves. Additionally, OD values were determined for the sh-ADM + sorafenib group (sorafenib concentration: 10 μmol/L) and sh-NC + sorafenib group to evaluate the impact of ADM knockdown on cellular sorafenib sensitivity.

### 2.10 Clonogenic survival assay

Cells from the sh-ADM group, sh-NC group, and sorafenib combination treatment groups were seeded in 6-well plates at a density of 500 cells per well, with three technical replicates established for each condition. Cells were maintained undisturbed in the incubator for a 2-week period. Upon completion of the culture period, culture medium was aspirated and cells were washed three times with ice-cold phosphate-buffered saline (PBS). Subsequently, cells were fixed with 4% paraformaldehyde for 15 min. After removal of the fixative, 0.1% crystal violet solution (Sigma) was applied for 30 min to stain the colonies. Excess staining solution was gently rinsed away with running water, and plates were air-dried before image acquisition using an Olympus optical microscope. Colonies were enumerated (colonies with diameter >0.1 mm were defined as valid), and colony formation rate was calculated accordingly.

### 2.11 Scratch wound healing assessment

Cells from the sh-ADM group, sh-NC group, and sorafenib combination treatment groups were seeded in 6-well plates and cultured until reaching 90% confluence. A uniform artificial “wound” was created by scratching the confluent cell monolayer perpendicular to the center line of the well bottom using a sterile 200 μL pipette tip. Cells were rinsed three times with PBS to remove detached cells, and culture medium was replaced with serum-free DMEM. Images of identical fields were captured using an Olympus optical microscope at 0 h and 24 h time points. Scratch width was quantified using ImageJ software, and the healing rate was calculated according to the following formula: Scratch healing rate (%) = [(Scratch width at 0 h - Scratch width at 24 h)/Scratch width at 0 h] × 100%.

### 2.12 Transwell migration and invasion assessments

For migration assessment, Transwell chambers (Corning, 8 μm pore size) were positioned in 24-well plates. The upper chamber received 100 μL of serum-free DMEM containing 5 × 10^4^ cells from the sh-ADM group, sh-NC group, or sorafenib combination treatment groups, while the lower chamber was filled with 600 μL of DMEM supplemented with 10% FBS. After a 24-h culture period, medium was discarded and non-migrated cells in the upper chamber were gently removed using a cotton swab. Cells were fixed with 4% paraformaldehyde for 15 min, followed by staining with 0.1% crystal violet for 30 min. Migrated cells were enumerated in 5 randomly selected fields under optical microscopy. For invasion assessment, the upper surface of Transwell chambers was pre-coated with Matrigel matrix (BD Biosciences, diluted 1:8) and incubated at 37 °C for 30 min to allow gel solidification. Subsequent procedural steps paralleled those of the migration assay: cells were seeded at a density of 1 × 10^5^ cells per well, cultured for 48 h, and invaded cells were then quantified.

### 2.13 Western blot protein analysis

Cells from the sh-ADM and sh-NC groups were harvested and lysed on ice for 30 min in RIPA lysis buffer supplemented with protease and phosphatase inhibitors (Beyotime Biotechnology). Following centrifugation at 12,000 rpm for 15 min at 4 °C, supernatants were collected as total protein extracts. Protein concentration was determined using a BCA protein assay kit (Beyotime Biotechnology). Protein samples (30 μg) were mixed with 5 × SDS loading buffer and denatured by boiling at 100 °C for 5 min. Protein separation was performed using 12% SDS-PAGE gels: electrophoresis was initiated at 80 V for 30 min, then increased to 120 V for an additional 90 min. Separated proteins were transferred to polyvinylidene difluoride (PVDF) membranes (Millipore) at a constant voltage of 100 V for 90 min. Membranes were blocked with 5% non-fat milk for 2 h, then incubated overnight at 4 °C with primary antibodies (ADM antibody, Abcam, 1:1000 dilution; β-actin antibody, Proteintech, 1:5000 dilution). The following day, membranes were washed three times with TBST (10 min per wash) and incubated with horseradish peroxidase (HRP)-conjugated secondary antibody (Jackson ImmunoResearch, 1:5000 dilution) for 1 h at room temperature. After three additional TBST washes, enhanced chemiluminescence (ECL) substrate (Millipore) was applied, and protein bands were visualized using a chemiluminescence imaging system (Tanon). Band intensity was quantified using ImageJ software.

### 2.14 Quantitative real-time polymerase chain reaction (qRT-PCR)

Total RNA was extracted from sh-ADM and sh-NC group cells using the RNAsimple Total RNA Extraction Kit (TIANGEN Biotech). RNA purity (A260/A280 ratio = 1.8–2.0) and concentration were assessed using a Nanodrop 2000 spectrophotometer (Thermo Fisher). One microgram of total RNA was reverse-transcribed into complementary DNA (cDNA) using the HiScript III 1st Strand cDNA Synthesis Kit with genomic DNA removal (Vazyme Biotech). qPCR reactions were conducted using NovoStart® SYBR qPCR SuperMix Plus (Novoprotein) in a 20 μL reaction system. The reaction mixture comprised: 2 μL cDNA template, 0.8 μL each of forward and reverse primers, 10 μL SYBR Mix, and 6.4 μL nuclease-free water. Thermal cycling conditions consisted of: initial denaturation at 95 °C for 30 s; followed by 40 cycles of denaturation at 95 °C for 10 s and annealing/extension at 60 °C for 30 s. GAPDH served as the internal reference gene, and relative ADM expression was calculated using the 2^−ΔΔCt^ method. Primer sequences are listed below:

**Table:** 

Gene	Forward primer (5′→3′)	Reverse primer (5′→3′)
ADM	TCC​CCC​TAT​TTT​AAG​ACG​TGA​ATG	CAT​GCA​CAC​AAA​CAC​ACT​CAC​AT
GAPDH	GCA​CCG​TCA​AGG​CTG​AGA​AC	TGG​TGA​AGA​CGC​CAG​TGG​A

### 2.15 Statistical analysis

All data processing, visualization, and statistical analyses were performed using R software (version 4.4.0) and GraphPad Prism (version 9.0). Correlations between continuous variables were evaluated using Spearman correlation coefficients. Prior to group comparisons, the Shapiro-Wilk test was employed to assess normality of data distribution. For data satisfying assumptions of normal distribution and homogeneity of variance, two-group comparisons utilized independent samples t-tests, while multi-group comparisons employed one-way analysis of variance (ANOVA). For data not meeting normal distribution assumptions or exhibiting heterogeneous variance, two-group comparisons utilized the Wilcoxon rank-sum test, and multi-group comparisons employed the Kruskal–Wallis H test. Categorical variables were compared using the chi-square (χ^2^) test. In scenarios involving multiple pairwise comparisons, the Benjamini–Hochberg method was applied to adjust the false discovery rate for control of Type I errors. All experiments were independently replicated three times to ensure reproducibility of results. Data are expressed as “mean ± standard deviation (SD)”.

## 3 Result

### 3.1 Characterization of hepatocellular carcinoma cells strongly correlated with TACE non-response phenotype and functional profiling

**FIGURE 1:**
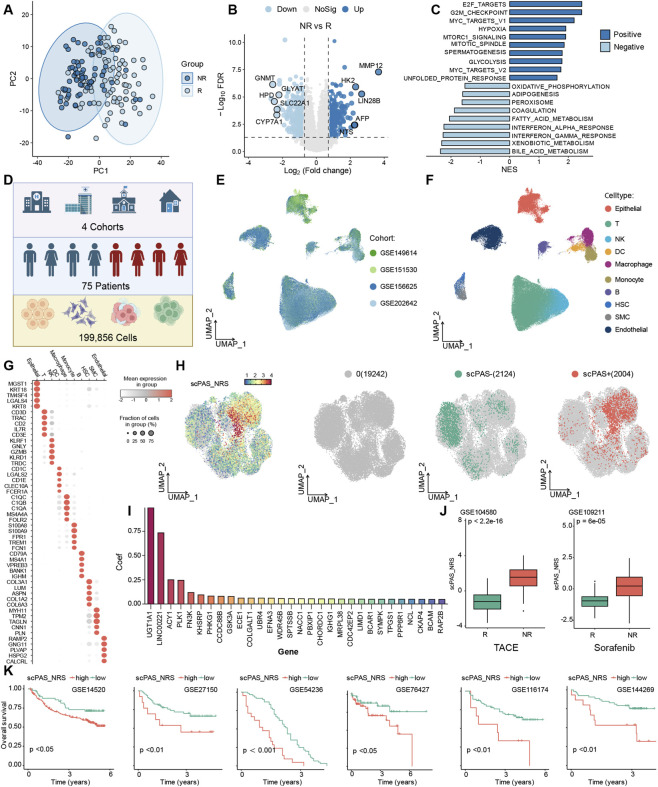
**(A)** Principal component analysis visualization demonstrating dimensional reduction of transcriptomic profiles from TACE-treated patient cohorts. **(B)** Comparative gene expression analysis contrasting TACE-responsive versus non-responsive patient populations. **(C)** Histogram representation of Gene Set Enrichment Analysis outcomes for genes exhibiting differential expression patterns. **(D)** Workflow illustration depicting single-cell transcriptomic data acquisition and cohort integration procedures. **(E)** Uniform Manifold Approximation and Projection (UMAP) visualizations displaying cellular clustering across four independent single-cell datasets. **(F)** UMAP-based dimensional reduction plots delineating distinct cellular population distributions. **(G)** Dot plot matrix illustrating signature gene expression patterns across characterized cell populations. **(H)** UMAP visualization of malignant cell populations stratified according to scPAS classification methodology. **(I)** Coefficient distribution histogram for gene variables incorporated within the scPAS predictive framework. **(J)** Comparative box plot distributions of scPAS scoring metrics in treatment-responsive versus treatment-refractory patient subgroups receiving TACE or sorafenib therapy. **(K)** Survival probability curves generated through Kaplan-Meier analysis comparing patients stratified by scPAS expression levels across multiple hepatocellular carcinoma cohorts.

To investigate transcriptional divergence between TACE-responsive and non-responsive patient cohorts, we initially employed PCA to examine gene expression profiles. The analysis revealed distinct clustering patterns between the two patient groups (Figure 1A). Differential gene expression analysis identified several significantly altered transcripts: non-responsive patients exhibited marked upregulation of MMP12, HK2, LIN28B, AFP, and NTS, alongside substantial downregulation of GNMT, GLYAT, HPD, SLC22A1, and CYP7A1 compared to responsive counterparts (Figure 1B). Functional interpretation of these key differentially expressed genes reveals important mechanistic insights. HK2, functioning as a glycolytic rate-limiting enzyme, demonstrates elevated expression in non-responsive patients, suggesting heightened glycolytic dependence consistent with the tumor Warburg effect. TACE-induced vascular embolization intensifies local ischemia and hypoxia, further constraining glycolytic metabolism and potentially contributing to therapeutic resistance. AFP, a classical hepatocellular proliferation marker, paradoxically shows elevated levels in non-responsive patients, suggesting that despite active proliferative status, these tumor cells possess mechanisms enabling survival under TACE-mediated ischemic stress and chemotherapeutic cytotoxicity, thereby evading apoptosis and manifesting treatment refractoriness. MMP12, predominantly engaged in extracellular matrix degradation and vascular reorganization, may indicate that elevated expression compromises effective vascular occlusion by TACE embolic materials in these patients’ tumor microenvironments, diminishing blood supply reduction efficacy while simultaneously augmenting tumor cell capacity for drug-resistant metastasis through matrix infiltration, collectively attenuating therapeutic outcomes. Gene Set Enrichment Analysis (GSEA) demonstrated that upregulated transcripts in non-responsive patients predominantly enriched in hypoxia and glycolysis pathways, whereas downregulated genes concentrated in oxidative phosphorylation and fatty acid metabolism pathways (Figure 1C). This signature distinctly illuminates fundamental metabolic reprogramming distinctions between patient cohorts. Despite elevated glycolytic gene expression, non-responsive patients may augment oxidative phosphorylation and fatty acid β-oxidation as compensatory metabolic routes to accommodate TACE-induced ischemic-hypoxic conditions, thereby tolerating treatment-associated metabolic perturbations. Conversely, responsive patients potentially lack such alternative metabolic adaptations, rendering them unable to counteract TACE-mediated metabolic suppression despite lower glycolytic gene expression, ultimately manifesting treatment sensitivity. These findings establish that hepatocellular carcinoma metabolic phenotypes constitute pivotal molecular determinants of TACE treatment response, with genes including HK2 and AFP serving as potential prognostic biomarkers for TACE sensitivity prediction. Elevated expression of these transcripts in non-responsive patients may herald suboptimal therapeutic outcomes.

Subsequently, we aggregated four hepatocellular carcinoma single-cell RNA sequencing datasets (GSE149614, GSE151530, GSE156625, and GSE202642) from multiple institutions, yielding 199,856 quality-controlled cells from 75 patients following rigorous filtering (Figures 1D,E). Leveraging established cellular markers, we delineated 10 distinct cell populations: Epithelial, T, NK, DC, Macrophage, Monocyte, B, HSC, SMC, and Endothelial cells (Figure 1F). Dot plot visualization illustrates marker gene expression across cell types (Figure 1G). To identify hepatocellular carcinoma cells strongly associated with the non-response (NR) phenotype, we isolated epithelial cells and implemented the scPAS algorithm integrated with TACE-responsive/non-responsive patient phenotypic data to construct a logistic regression classifier for tumor cell categorization, distinguishing two phenotypes: scPAS+ (non-responsive) and scPAS- (responsive) (Figure 1H). Within this logistic regression framework, UGT1A1 and LINC00221 emerged as top-scoring discriminatory genes (Figure 1I). LINC00221, a long non-coding RNA, demonstrates crucial discriminatory capacity in scPAS+/-phenotype classification with substantial mechanistic implications. Although hepatocellular carcinoma research on LINC00221 remains limited, functionally analogous LINC00961 influences tumor chemoresistance through glucose metabolism-related gene regulation or hypoxia-inducible factor pathway modulation. Extrapolating from current findings, LINC00221 may participate in TACE non-response phenotype development through dual mechanisms: first, preferential expression in scPAS + cells may suppress glycolytic pathway activity while activating fatty acid metabolism-associated genes, facilitating tumor cell adaptation to TACE-induced ischemic conditions; second, enhancement of tumor cell invasive capacity through epithelial-mesenchymal transition-related gene regulation, diminishing TACE cytotoxic efficiency against tumor cells.

To validate clinical significance of these phenotypes, we examined correlations with patient clinical characteristics across multiple datasets. Analysis revealed that in GSE104580 (TACE-treated cohort) and GSE109211 (sorafenib-treated cohort), the scPAS + phenotype exhibited preferential expression in non-responsive patients (Figure 1J), indicating strong association with TACE treatment responsiveness. Kaplan-Meier survival analysis further confirmed that across GSE14520, GSE27150, GSE54236, GSE76427, GSE116174, and GSE144269 datasets, patients harboring the scPAS + phenotype demonstrated significantly abbreviated overall survival compared to scPAS- phenotype patients (Figure 1K), establishing scPAS + as a potential adverse prognostic indicator for hepatocellular carcinoma.

### 3.2 Identification of gene modules strongly correlated with scPAS + cells through hdWGCNA analysis

**FIGURE 2:**
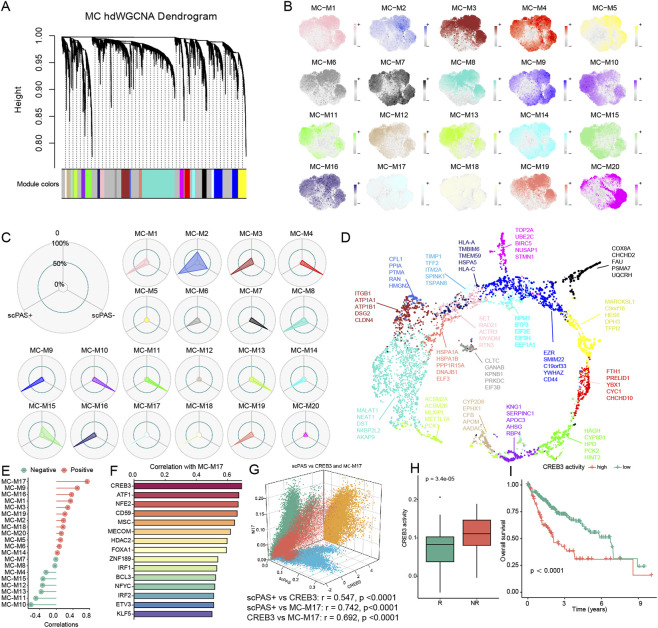
**(A)** Transcriptomic modularization of malignant cell populations through weighted gene co-expression network analysis implementation. **(B)** Spatial distribution patterns of twenty identified gene co-expression modules visualized within single-cell resolution datasets. **(C)** Modular preference mapping demonstrating association strength between twenty gene networks and scPAS phenotypic classifications. **(D)** Core gene constituents defining the functional architecture of twenty co-expression modules. **(E)** Univariate correlation matrix analysis quantifying relationships between modular gene networks and scPAS cellular stratification. **(F)** Bar chart representation depicting correlation coefficients between computationally predicted upstream transcriptional regulators and the scPAS + M17 co-expression module. **(G)** Three-dimensional correlation scatter visualization integrating scPAS + cellular expression signatures, M17 module transcriptional activity, and CREB3 regulatory dynamics. **(H)** Comparative box plot analysis of CREB3 transcription factor activation status in TACE treatment-responsive versus treatment-resistant patient populations. **(I)** Survival trajectory curves employing Kaplan-Meier methodology to compare patient cohorts stratified by CREB3 transcriptional activity levels.

To comprehensively dissect gene expression architectures and functional attributes of scPAS + cells, we partitioned single-cell transcriptomic data into gene modules via high-dimensional weighted gene co-expression network analysis (hdWGCNA), successfully identifying 20 co-expression modules with spatial expression localization elucidating distribution patterns within tumor tissues (Figures 2A,B). Module-phenotype correlation analysis revealed 11 modules (M1, M2, M3, M6, M8, M9, M14, M16, M17, M18, M19) demonstrating preferential expression in scPAS + cells, whereas modules M4, M7, M10, M11, M13, and M15 exhibited enrichment in scPAS- cells, underscoring substantial molecular functional divergence between cell types (Figure 2C). Core constituents of the M1 gene module encompass HSPA1A, HSPA1B, PPP1R15A, DNAJB1, and ELF3, collectively representing heat shock protein family members and associated regulatory genes. HSPA1A/HSPA1B, as pivotal HSP70 family members, not only preserve protein homeostasis under stress conditions through chaperone function but also suppress tumor cell apoptosis via PI3K/Akt pathway activation, concordant with scPAS + cell survival advantages within tumor hypoxic and nutrient-depleted microenvironments. PPP1R15A, a critical endoplasmic reticulum stress pathway component, attenuates stress-induced damage through eIF2α dephosphorylation regulation, further reinforcing scPAS + cell environmental adaptability. ELF3, an epithelium-specific transcription factor, may synergize with HSPs to maintain scPAS + cell epithelial phenotype, preventing excessive stress-triggered epithelial-mesenchymal transition.

The M17 gene module comprises core genes including TIMP1, TFF2, ITM2A, SPINK1, and TSPAN8, intimately associated with tumor stemness maintenance and invasion-metastasis capacity. Although TIMP1 functions as a matrix metalloproteinase inhibitor capable of restraining MMP-mediated matrix degradation, contemporary studies confirm its capacity to activate PI3K/Akt signaling through CD63 binding, promoting hepatocellular carcinoma stem cell self-renewal. TSPAN8, a tetraspanin family member, augments cellular basement membrane adhesion through integrin α6β4 localization regulation, providing anchorage sites for tumor cell vascular invasion. SPINK1 mitigates tumor cell apoptosis caused by excessive protease activation through trypsin-like serine protease activity inhibition, with elevated expression significantly correlating with increased vascular invasion and recurrence risk in hepatocellular carcinoma patients. Coordinated expression of these genes suggests the M17 module may constitute the core functional unit positioning scPAS + cells as “tumor seed cells.” The M20 gene module primarily comprises TOP2A, UBE2C, and STMN1, principally regulating cell proliferation and promoting hepatocellular carcinoma growth (Figure 2D). Univariate correlation analysis demonstrated highest correlation coefficients between M17 and M9 modules with scPAS + cell expression patterns (Figure 2E).

Furthermore, we quantified transcription factor activity utilizing the SCENIC algorithm. Correlation analysis identified CREB3 (cAMP response element-binding protein 3) activity as most strongly correlated with M17 module expression (Figure 2F). As an endoplasmic reticulum stress-responsive transcription factor, CREB3 regulates HSP and cyclin gene expression through CRE element binding in gene promoter regions, highly consistent with M17 module function. Three-dimensional correlation scatter plot validation confirmed significant positive correlations among scPAS + cell expression characteristics, M17 module gene expression levels, and CREB3 transcription factor activity, establishing CREB3 as a putative core transcription factor governing scPAS + cell molecular characteristics (Figure 2G). Clinical specimen analysis revealed significantly elevated CREB3 transcription factor activity in TACE non-responsive patients (Figure 2H), with Kaplan-Meier survival analysis demonstrating markedly shortened median survival in patients with elevated CREB3 transcription factor activity compared to low-expression counterparts (Figure 2I). These findings suggest CREB3-mediated scPAS + cell functional activation may represent a critical mechanism underlying TACE treatment resistance and adverse patient prognosis. TACE-induced tumor localized ischemia and hypoxia trigger CREB3 activation, subsequently upregulating M17 module (stemness/metastasis) gene expression, enabling scPAS + cells to tolerate treatment-induced damage while maintaining tumor progression capacity.

### 3.3 Elucidating scPAS + cell roles in immune microenvironment architecture through cell-cell communication analysis

**FIGURE 3:**
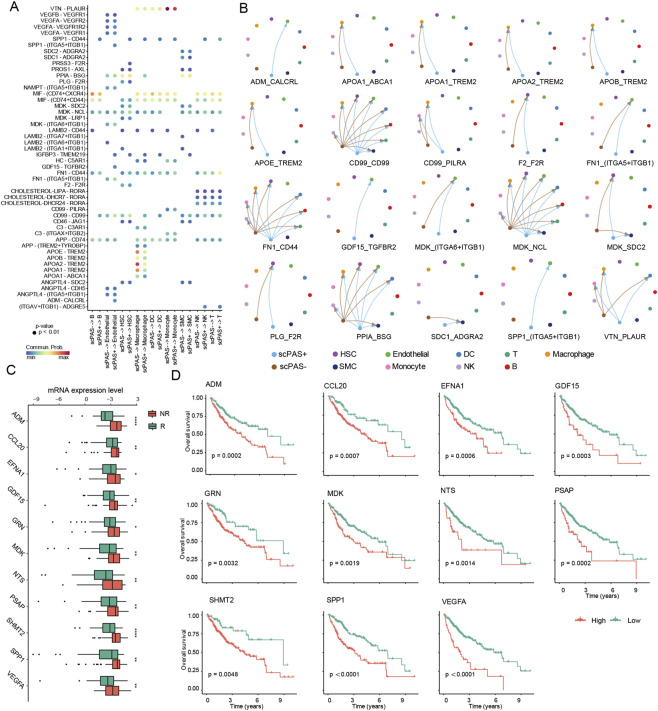
**(A)** Intercellular communication network analysis elucidating signaling interaction architectures between scPAS + cellular populations and diverse tumor microenvironment constituents. **(B)** Circular chord diagram visualization contrasting differential intercellular communication patterns between scPAS+ and scPAS- cell populations. **(C)** Distribution box plots comparing expression magnitudes of eleven ligand-encoding genes between TACE treatment-responsive and treatment-refractory patient cohorts. **(D)** Kaplan-Meier survival probability curves stratifying patients according to composite expression levels of eleven ligand genes.

To delineate core mechanisms whereby scPAS + cells influence TACE treatment response in hepatocellular carcinoma patients through immune microenvironment modulation, we employed cell-cell communication analysis to resolve signal interaction networks between scPAS + cells and other tumor microenvironment constituents (endothelial cells, immune cells, stellate cells, etc.). We discovered that scPAS+ and scPAS- cells construct targeted regulatory networks for tumor microenvironment (TME) cells through differential ligand expression including ADM, CCL20, EFNA1, GDF15, GRN, MDK, NTS, PSAP, SHMT2, SPP1, and VEGFA, with substantial ligand expression profile divergence between cell types (Figure 3A). Cell type-specific analysis revealed that compared to scPAS- cells, scPAS + cells exhibited significantly amplified regulatory effects on endothelial cells, with core regulatory molecules comprising ADM, SPP1, and FN1, suggesting scPAS + cells may remodel tumor vascular microenvironments through endothelial cell targeting (Figure 3B). ADM, a hypoxia stress-responsive ligand, demonstrates elevated expression in scPAS + cells highly concordant with post-TACE tumor localized ischemic microenvironments. Through CLR/RAMP2 receptor complex binding on endothelial cell surfaces, ADM activates cAMP/PKA signaling cascades, simultaneously promoting endothelial cell survival and inducing vascular endothelial growth factor receptor phosphorylation, accelerating angiogenesis in ischemic regions. This process counteracts TACE treatment “vascular blockade” effects, providing nutrient supply for residual scPAS + cells, directly precipitating treatment resistance. SPP1 exhibits dual “vascular regulation” and “immune suppression” functionalities. At the vascular level, SPP1 enhances extracellular matrix (ECM)-endothelial cell adhesion through integrin αvβ3 binding on endothelial cells, promoting vascular maturation and stability. At the immune level, it recruits myeloid-derived suppressor cells (MDSCs) to tumor regions, inhibiting CD8^+^ T cell cytotoxic activity, forming synergistic “vascular protection-immune evasion” effects, further consolidating scPAS + cell survival advantages. FN1, as a core ECM component secreted by scPAS + cells, enhances scPAS + cell-vascular wall anchoring through “endothelial cell-tumor cell” adhesion bridge construction, reducing tumor cell detachment and apoptosis following TACE treatment. Simultaneously, FN1 activates PI3K/Akt pathways in endothelial cells, upregulating matrix MMP9 expression and promoting vascular wall remodeling, providing channels for tumor cell invasion and metastasis. Comparative ligand expression analysis between TACE-responsive and non-responsive patients demonstrated significant overexpression of all 11 ligands in non-responsive patients (all P < 0.01), with ADM exhibiting the most pronounced expression differential (Figure 3C). This finding establishes ADM as a core “hypoxia-angiogenesis” axis molecule, with elevated expression serving as an early warning biomarker for TACE resistance. Post-TACE ischemic stress induces robust ADM secretion from scPAS + cells, rapidly initiating vascular repair processes, leading to therapeutic efficacy attenuation. Kaplan-Meier survival analysis revealed significantly abbreviated median overall survival in patient cohorts with elevated combined expression of the 11 ligands compared to low-expression groups (Figure 3D). The underlying mechanism likely involves ADM-mediated sustained angiogenesis providing stable blood supply for tumors, promoting scPAS + cell proliferation and dissemination.

### 3.4 Construction of TACE responsiveness prediction model based on module genes and core gene identification

**FIGURE 4:**
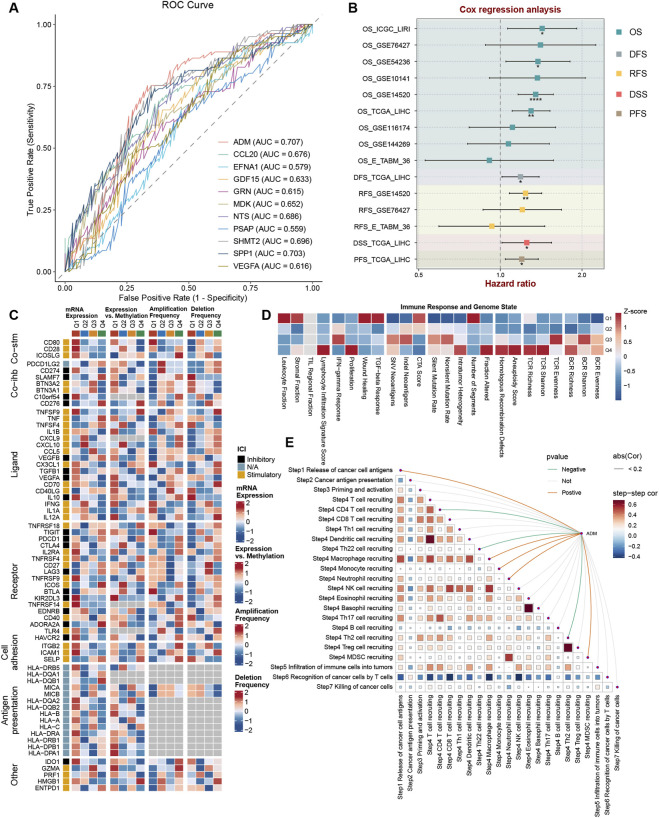
**(A)** Receiver operating characteristic curve analysis quantifying diagnostic performance metrics for TACE treatment response prediction in patient populations. **(B)** Forest plot representation of univariate Cox proportional hazards regression analysis examining ADM expression impact on clinical outcomes across multiple hepatocellular carcinoma datasets. **(C)** Integrated heatmap visualization displaying transcriptional abundance, epigenetic methylation status, genomic amplification events, and deletion frequencies of immune-regulatory molecules across patient quartiles stratified by ADM expression magnitude. **(D)** Comprehensive heatmap profiling immune microenvironment landscape characteristics across patient subgroups categorized by ADM expression quartile rankings. **(E)** Correlation heatmap matrix depicting ADM association patterns across the sequential seven-stage tumor immune response cascade.

To further identify core genes with optimal predictive value for hepatocellular carcinoma patient TACE treatment responsiveness, we initially evaluated sensitivity and specificity of 11 ligand genes demonstrating most significant differential expression between scPAS+/-cells in predicting patient TACE responsiveness through receiver operating characteristic (ROC) curve analysis. Results demonstrated ADM exhibited superior predictive accuracy with an area under the curve (AUC) value of 0.707, significantly exceeding other ligand genes, establishing ADM as a core candidate gene regulating TACE responsiveness (Figure 4A). To validate ADM prognostic value, univariate Cox regression analysis across multiple cohorts identified elevated ADM expression as an independent risk factor for abbreviated overall survival in hepatocellular carcinoma patients (Figure 4B). Subsequently, we stratified patients into four quartile groups (Q1: highest to Q4: lowest) according to ADM expression levels, analyzing molecular and immune characteristic differences among groups. With progressive ADM expression decline, transcriptional levels of pro-tumorigenic inflammatory cytokines IL1B and CX3CL1 demonstrated gradual downregulation, suggesting elevated ADM expression may accelerate tumor progression through pro-inflammatory microenvironment enrichment. Concurrently, methylation levels of CD70 and CD40LG, critical co-stimulatory molecules for T cell activation, synchronously decreased. Methylation inhibition relief signifies transcriptional activation capability, potentially enhancing T cell activation signals. Additionally, amplification levels of CXCL9 and CXCL10 genes with CD8^+^ T cell-specific chemotactic effects significantly increased, with tumor necrosis factor gene deletion frequency progressively decreasing. These dynamic molecular-level alterations clearly indicate elevated ADM expression strongly correlates with attenuated tumor microenvironment immune responses (Figure 4C).

In-depth immune landscape analysis revealed that although BCR and TCR repertoire abundance and diversity in high ADM expression patients significantly exceeded low-expression cohorts, with increased stromal cell numbers and total lymphocyte infiltration, synchronous upregulation of TGF-β signaling pathway, epithelial-mesenchymal transition (EMT), and wound healing-related pathway expression formed an “EMT-immune suppression” synergistic effect, suggesting high ADM expression patients possess both enhanced metastatic potential and immune evasion capacity (Figure 4D). In tumor immune response seven-step cascade analysis, ADM expression positively correlated with tumor antigen generation and presentation, indicating promotion of malignant tumor cell proliferation and tumor-associated antigen production, while negatively correlating with CD4^+^ T cell and Th22 cell recruitment efficiency. Conversely, significant positive correlations existed with myeloid cell infiltration degree including MDSCs, tumor-associated macrophages (TAMs), and neutrophils (Figure 4E). This result explains the paradoxical phenomenon of “increased total lymphocyte infiltration but decreased TCR functional activity” in high ADM expression patients—massive myeloid cell infiltration constructs immunosuppressive microenvironments, offsetting T cell anti-tumor effects. MDSCs suppress T cell proliferation and cytotoxicity through IL-10 and TGF-β secretion, while TAMs block T cell recognition through tumor antigen phagocytosis, ultimately forming an immune paralysis state of “enhanced antigen presentation but ineffective immune killing,” with ADM serving as the critical regulatory hub of this pathological process.

### 3.5 ADM promotes hepatocellular carcinoma cell proliferation, migration, and invasion

**FIGURE 5:**
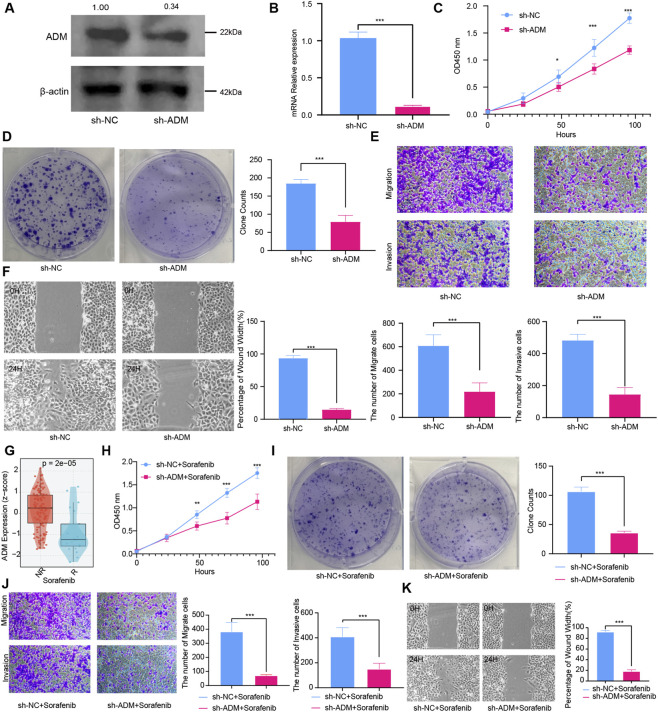
**(A)**. Western blot quantification of ADM protein abundance in stable knockdown (sh-ADM) versus negative control (sh-NC) cellular populations. **(B)** Quantitative PCR measurements of ADM transcript levels normalized between sh-ADM and sh-NC experimental groups. **(C)** Temporal optical density measurements at 450 nm wavelength from Cell Counting Kit-8 proliferation assays comparing sh-ADM and sh-NC populations. **(D)** Photographic documentation and quantification of clonogenic survival capacity in sh-ADM versus sh-NC cellular cohorts. **(E)** Enumeration of translocated and matrix-invasive cells in sh-ADM and sh-NC experimental conditions. **(F)** Quantitative assessment of scratch wound closure percentage at 24-h timepoint in sh-ADM versus sh-NC groups. **(G)** Distribution box plot illustrating ADM expression magnitudes stratified by sorafenib treatment response status within the GSE109211 patient cohort. **(H)** Optical density readings at 450 nm from Cell Counting Kit-8 assays evaluating proliferation in combined sh-ADM + sorafenib versus sh-NC + sorafenib treatment groups. **(I)** Comparative clonogenic capacity visualization in combined sh-ADM + sorafenib versus sh-NC + sorafenib experimental conditions. **(J)** Quantification of migratory and invasive cellular populations in combined sh-ADM + sorafenib versus sh-NC + sorafenib treatment paradigms. **(K)** Scratch wound healing rate determination at 24-h endpoint in combined sh-ADM + sorafenib versus sh-NC + sorafenib experimental groups.

To investigate ADM’s influence on hepatocellular carcinoma cell proliferation, migration, and invasion, we established an Huh7 cell line with ADM knockdown. Substantial reductions in both ADM protein levels and mRNA expression were confirmed (Figures 5A,B), validating efficient ADM silencing. CCK-8 assay results demonstrated that in Huh7 cells, the sh-ADM group exhibited markedly decreased proliferation rates compared to the sh-NC group (Figure 5C). Similarly, colony formation experiments indicated significantly diminished clonogenic capacity in the sh-ADM group relative to the sh-NC group (Figure 5D). These collective findings establish that ADM downregulation suppresses hepatocellular carcinoma cell proliferation. Wound healing and Transwell migration assays revealed that in Huh7 cells, the sh-ADM group demonstrated significantly reduced migration rates compared to the sh-NC group (Figures 5E,F), indicating ADM expression reduction impairs hepatocellular carcinoma cell migratory capacity. Furthermore, Transwell invasion assays performed in Huh7 cells showed fewer cells in the sh-ADM group traversed the chamber relative to the sh-NC group, establishing that ADM downregulation attenuates hepatocellular carcinoma cell invasive capacity (Figure 5E).

### 3.6 ADM promotes sorafenib resistance in hepatocellular carcinoma

We further analyzed ADM expression in patients with differential sorafenib responses (responsive vs. non-responsive) utilizing the GSE109211 dataset. Results demonstrated patients with elevated ADM expression failed to achieve treatment response (Figure 5G), suggesting elevated ADM expression may serve as a potential biomarker for sorafenib resistance. This resistance mechanism likely associates with ADM’s roles in promoting angiogenesis and sculpting immunosuppressive microenvironments: ADM activates the VEGFR2 pathway to counteract sorafenib’s inhibitory effects on endothelial cells while simultaneously recruiting MDSCs to diminish drug-mediated tumor cell cytotoxicity. CCK-8 assays demonstrated that proliferation rates in the sh-ADM + sorafenib group were significantly lower than the sh-NC + sorafenib group (Figure 5H). Colony formation experiments revealed the sh-ADM + sorafenib group formed substantially fewer colonies than the sh-NC + sorafenib group (Figure 5I), confirming ADM knockdown markedly enhances sorafenib’s capacity to inhibit hepatocellular carcinoma cell proliferation. Wound healing and Transwell migration assays revealed 24-h migration rates in the sh-ADM + sorafenib group were significantly reduced compared to the sh-NC + sorafenib group (Figures 5J,K). Additionally, Transwell invasion assays indicated fewer cells in the sh-ADM + sorafenib group crossed the membrane compared to the sh-NC + sorafenib group (Figure 5J). These findings suggest ADM-targeted therapy combined with sorafenib may represent a novel precision therapeutic strategy for hepatocellular carcinoma, particularly for sorafenib-resistant patients with elevated ADM expression, providing innovative avenues to enhance targeted therapy efficacy.

## 4 Discussion

TACE is presently endorsed as a frontline therapeutic approach for intermediate-to-advanced hepatocellular carcinoma (HCC) in contemporary clinical guidelines, yet its therapeutic benefits remain substantially constrained by treatment resistance rates reaching 40%–60% (Raoul et al., 2019; Martin et al., 2020). Clinical evidence demonstrates that approximately half of patients undergoing standardized TACE experience disease progression within 6–12 months post-intervention, with median overall survival merely extending to 15–20 months. This clinical reality not only profoundly compromises patient quality of life but also represents a fundamental obstacle impeding therapeutic advancement for intermediate-to-advanced HCC (Sangro et al., 2025; Chen et al., 2023). The multifaceted nature of resistance mechanisms extends far beyond intrinsic tumor cell tolerance, encompassing intricate dynamic interactions among diverse tumor microenvironment (TME) components and cross-resistance phenomena spanning multiple therapeutic modalities (Myojin et al., 2025). These complexities have historically eluded systematic investigation, presenting formidable challenges for formulating clinically precise intervention strategies. Leveraging single-cell RNA sequencing technology integrated with high-dimensional weighted gene co-expression network analysis (hdWGCNA), cell-cell communication network mapping, and functional validation, our investigation represents the first comprehensive delineation of a TACE resistance molecular landscape centered on scPAS + cells at single-cell resolution. We have elucidated ADM’s dual regulatory function as a “central hub molecule” in therapeutic resistance while simultaneously unveiling the pivotal mediating role of transcription factor CREB3 in orchestrating “stemness preservation-stress adaptation” mechanisms. These discoveries not only address critical knowledge gaps in existing TACE resistance research but also establish translatable theoretical foundations and actionable therapeutic targets for precision management of intermediate-to-advanced HCC.

Our investigation successfully identified the scPAS + cell population strongly associated with the TACE non-response (NR) phenotype utilizing scPAS methodology. This cellular subset exhibits characteristic overexpression of molecular signatures including LINC00221, HK2, and AFP, alongside pronounced enrichment of pathways governing GLYCOLYSIS, HYPOXIA, and epithelial-mesenchymal transition (EMT). On one front, HK2, serving as a critical rate-limiting enzyme in glycolytic metabolism, confers upon scPAS + cells a quintessential “Warburg effect” wherein they preferentially utilize glycolysis for energy generation even under aerobic conditions. This demonstrates substantial synergy with the AKR1B1-orchestrated “glucose-lipid-glutathione” metabolic network (Wang et al., 2025; Li et al., 2025). AKR1B1 furnishes glycolytic substrates through lipid remodeling regulation, while HK2 sustains cellular energy provision by amplifying glycolytic efficiency, collectively forming the metabolic resistance foundation of scPAS + cells. Conversely, scPAS + cells can rapidly transition metabolic programs within TACE-induced ischemic-hypoxic microenvironments by activating fatty acid β-oxidation and oxidative phosphorylation pathways, circumventing energy crises precipitated by glycolytic inhibition. This “metabolic plasticity” constitutes a defining characteristic enabling their tolerance to TACE intervention. Notably, our investigation represents the first documentation of the long non-coding RNA LINC00221s critical discriminatory function in scPAS + cells. Although LINC00221 remains unreported in HCC contexts, its functionality may parallel lncRNA ZFAS1, which mediates donafenib resistance through LSD1/CoREST/p65 axis activation (Hou et al., 2024). We hypothesize LINC00221 may participate in resistance through dual mechanisms: first, functioning as a “molecular sponge” sequestering glycolysis-related microRNAs, thereby relieving transcriptional suppression of HK2; second, interacting with transcription factors such as CREB3 to govern EMT-related gene expression, augmenting scPAS + cell invasive capacity. This hypothesis warrants rigorous experimental validation in subsequent investigations. Furthermore, AFP—highly expressed in scPAS + cells—extends beyond its conventional role as a clinical HCC diagnostic marker. Our findings suggest it may suppress tumor cell apoptosis through PI3K/Akt pathway activation. AFP can engage insulin-like growth factor receptors, mimicking insulin-like growth factor functions and promoting anti-apoptotic protein Bcl-2 expression, enabling scPAS + cells to withstand TACE-mediated ischemic injury and chemotherapeutic cytotoxicity. This discovery supplements AFP’s non-canonical functions in the “HCC proliferation-drug resistance” paradigm, harmonizing perfectly with conclusions that scPAS + cells enhance stress tolerance via p53 and HIF-1 pathways (Chen et al., 2023; Liu and Zhou, 2025).

At the transcriptional regulatory stratum, our investigation confirmed through SCENIC algorithm implementation that CREB3 constitutes the core transcription factor governing the M17 module. This finding provides specific molecular explanation for CREB3’s “dual functionality” in TACE resistance contexts: previous research demonstrated CREB3 can not only suppress AKT signaling through competitive insulin receptor binding, exerting tumor-suppressive effects (He et al., 2024), but also mediate donafenib resistance by transcriptionally activating ZFAS1 (Hou et al., 2024). Our study further established that within TACE-induced ischemic-hypoxic microenvironments, CREB3 undergoes activation by endoplasmic reticulum stress signals, subsequently upregulating M17 module gene expression through binding cAMP response elements (CRE) in their promoter regions. TIMP1 activates PI3K/Akt pathways via CD63 binding, preserving scPAS + cell stemness characteristics (Xu et al., 2025); TSPAN8 amplifies cellular basement membrane adhesion capacity by regulating integrin α6β4 membrane localization, providing anchorage sites for tumor cell vascular invasion; SPINK1 inhibits trypsin-like serine protease activity, mitigating tumor cell apoptosis from excessive protease activation, with its overexpression significantly correlating with increased vascular invasion and recurrence risk in HCC patients (Li et al., 2025). Cell-cell communication network analysis unveiled synergistic resistance mechanisms between scPAS + cells and TME constituents. Through analyzing signal interactions between scPAS + cells and endothelial/immune cells, we discovered that signaling axes comprising ligands including ADM, SPP1, FN1, CCL20, and GDF15 form the regulatory core of TME functions. Among these, ADM—as a pivotal ligand secreted by scPAS + cells—demonstrates overexpression significantly associated with TACE non-response phenotypes while attenuating TACE therapeutic efficacy through dual “angiogenesis-immune suppression” mechanisms.

At the vascular dimension, ADM engages CLR/RAMP2 receptor complexes on endothelial cell surfaces, activating cAMP/PKA signaling cascades: promoting endothelial survival through Bcl-2 upregulation while inducing VEGFR2 phosphorylation, accelerating angiogenesis in ischemic territories. This directly counteracts TACE’s core “vascular embolization” effect, providing nutrient supply for residual scPAS + cells. Our investigation also revealed ADM-mediated angiogenesis exhibits “targeting specificity”: predominantly promoting microvessel formation within and surrounding tumors while minimally affecting normal hepatic tissue vasculature. This may relate to elevated HIF-1α concentrations in tumor microenvironments, consistent with conclusions that ADM promotes tumor angiogenesis through HIF-1 signaling pathways (Liu and Zhou, 2025). At the immune stratum, ADM recruits MDSCs and TREM2-positive tumor-associated macrophages (TAMs) to tumor regions, suppressing CD8^+^ T cell cytotoxic activity. ADM binding to CLR receptors on MDSC surfaces activates STAT3 pathways, promoting MDSC secretion of immunosuppressive factors including IL-10 and TGF-β. Concurrently, ADM can induce TREM2+ TAMs to release Galectin-1, reducing CXCL9 production thereby inhibiting CD8^+^ T cell tumor recruitment (Zhang et al., 2025). Additionally, Liu and colleagues confirmed ADM positively correlates with immune checkpoint genes including PD-L1 and CD276 (Sangro et al., 2025). Significantly, our investigation also established ADM as a critical marker for cross-resistance between TACE and sorafenib. In the GSE109211 cohort, sorafenib non-response rates in patients with elevated ADM expression substantially exceeded those with low expression; *in vitro* and *in vivo* experiments demonstrated ADM knockdown markedly enhances sorafenib’s inhibitory effects on HCC cell proliferation. This finding provides crucial evidence for adjusting cross-treatment strategies in clinical practice for high ADM expression patients, suggesting such patients may benefit more from combined anti-ADM therapy rather than sorafenib monotherapy (Xuan et al., 2025).

SPP1 and FN1 also fulfill pivotal roles in scPAS + cell-mediated TME regulation. As a “resistance mediator,” SPP1 functionality in our study transcends previously reported “immune barrier construction” (Xu et al., 2025; Seyhan et al., 2025); it additionally remodels vascular microenvironments and extracellular matrix (ECM) through interactions with endothelial cells and hepatic stellate cells (HSCs). At the vascular level, SPP1 binds integrin αvβ3 on endothelial surfaces, strengthening ECM-vascular endothelial adhesion and promoting vascular maturation and stability. This renders tumor blood supply more resistant to TACE embolic blockade; meanwhile, SPP1 activates PI3K/Akt pathways in endothelial cells, upregulating matrix metalloproteinase expression and facilitating vascular wall remodeling, establishing channels for tumor cell invasion and metastasis. At the immune level, SPP1 binds CD44 on T cell surfaces, inhibiting T cell proliferation and IFN-γ production (Tan et al., 2022). Furthermore, spatial transcriptomics research by Tong and colleagues demonstrated tumor-derived SPP1 activates PI3K/Akt pathways in HSCs, promoting their differentiation into cancer-associated fibroblasts (CAFs) (Tong et al., 2024). As a core ECM component, FN1—following secretion by scPAS + cells and CAFs—participates in resistance through dual mechanisms: first, constructing “endothelial-tumor cell” adhesion bridges whereby FN1 simultaneously binds integrin α5β1 on endothelial surfaces and integrin αvβ3 on tumor cell surfaces, enhancing scPAS + cell vascular wall anchoring and reducing tumor cell detachment and apoptosis following TACE treatment; second, activating PI3K/Akt pathways in endothelial cells, upregulating MMP9 expression and promoting vascular wall remodeling, providing invasion and metastasis channels. This complements mechanisms whereby CAF-derived FN1 promotes pancreatic cancer metastasis (Zhu et al., 2025; Guerrero-Barberà et al., 2024), suggesting FN1’s metastasis-promoting effects exhibit pan-cancer universality. Additionally, our investigation revealed ligands including CCL20 and GDF15 fulfill auxiliary roles in scPAS + cell-mediated TME regulation, with these ligands’ synergistic effects further refining the molecular network governing scPAS + cell TME modulation.

Immune microenvironment dynamic regulation represents another critical dimension of TACE resistance. Our investigation discovered that in TMEs of high ADM expression patients, myeloid cell infiltration including MDSCs and TREM2+ TAMs substantially increases while CD4^+^ T cell and Th22 cell recruitment diminishes, forming an “immune paralysis” state. Even with increased B cell receptor (BCR) and T cell receptor (TCR) repertoire abundance and diversity, T cell cytotoxic function remains suppressed by myeloid cell-secreted IL-10 and TGF-β. This outcome highly aligns with conclusions that “dynamic myeloid populations predict TACE response.” Ren and colleagues found pretreatment monocyte-derived MDSC levels predict TACE efficacy, with mMDSCs suppressing immunity through arginase and reactive oxygen species (ROS) pathways (Ren et al., 2025; Yue et al., 2024). Our study further clarifies that scPAS + cells regulate mMDSC infiltration and function through ligands including ADM, establishing a regulatory cascade of “scPAS + cells - myeloid cells - immune suppression,” supplementing specific upstream signals for myeloid cell involvement in TACE resistance. Additionally, the CXCL9/SPP1 ratio predicts immunotherapy response (Gu et al., 2025); high SPP1 secretion by scPAS + cells in our study may reduce CXCL9/SPP1 ratios, suggesting high ADM expression patients may benefit from combined CXCL9 agonist or SPP1 inhibitor treatment to reverse immunosuppressive microenvironments.

From clinical translation perspectives, our findings hold multifaceted value. First, as a dual predictive marker for TACE and sorafenib resistance, ADM detection enables pre-treatment patient stratification: high ADM expression patients may prioritize combined anti-ADM therapy over TACE or sorafenib monotherapy. Second, scPAS + cell phenotypes and CREB3 activity serve as prognostic evaluation indicators, assisting clinicians in determining patient recurrence risk and survival expectations. Finally, the “ADM targeting + TACE + sorafenib” triple strategy proposed herein complements standard “TACE + immune checkpoint inhibitor + anti-angiogenic drug” regimens; particularly for cross-resistant patients with elevated ADM expression, simultaneous blockade of angiogenesis, tumor stemness inhibition, and immune suppression reversal may achieve therapeutic breakthroughs (Rimassa et al., 2023; Abou-Alfa et al., 2022; Zheng et al., 2021).

Our investigation possesses limitations requiring subsequent work: clinical agents targeting ADM have not undergone clinical trials in HCC contexts, with combination TACE safety and efficacy requiring exploration. Future investigations may emphasize synergistic inhibition of ADM alongside other resistance targets, or combine with personalized neoantigen vaccines to further improve TACE-resistant patient prognosis (D’Alessio and Rimassa, 2025; Desert et al., 2025). In summary, through multidimensional technical approaches, our investigation unveils molecular networks whereby scPAS + cells construct TACE resistance through metabolic reprogramming, transcriptional regulation, and microenvironment interactions, particularly clarifying ADM’s central position as a “hub molecule” in resistance prediction and therapeutic targeting. These discoveries not only supplement existing theoretical frameworks for HCC TACE resistance mechanisms but also provide actionable markers and targets for formulating precise clinical intervention strategies, promising to advance intermediate-to-advanced HCC treatment transformation from “empirical” to “precision-guided” paradigms.

## Data Availability

Transcriptomic and clinical data for all patients were obtained from TCGA and GEO databases. The accession numbers can be found in the article.
