# Supplementary figures and images for: Adrenomedullin orchestrates treatment resistance in hepatocellular carcinoma via immune microenvironment remodeling
Source: Front Genet. 2025 Dec 11;16:1721263. doi: 10.3389/fgene.2025.1721263 (PMC12736389; doi:10.3389/fgene.2025.1721263)

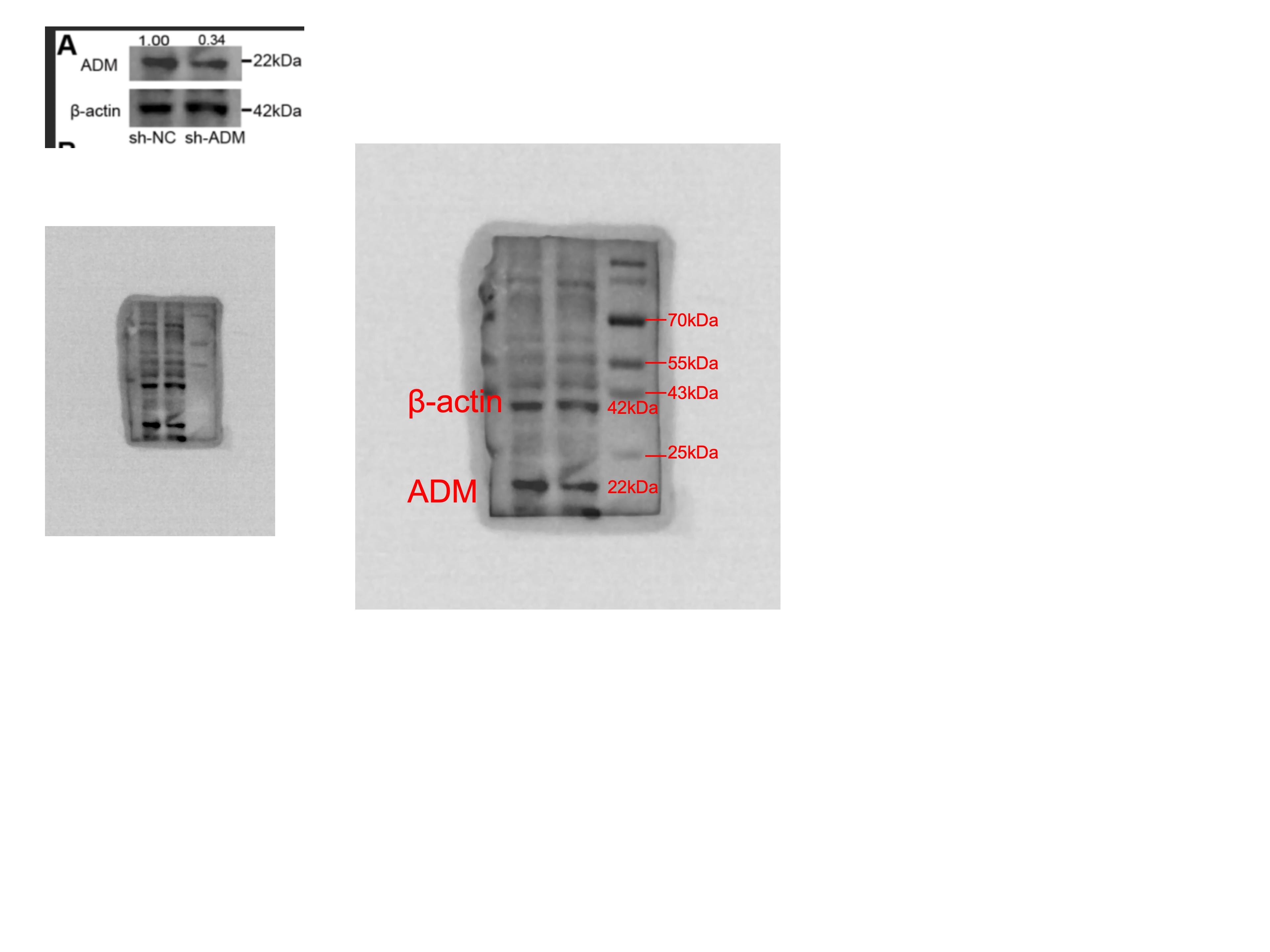

Supplement: Supplementary file 1 [file Image1.jpeg]
